# Efficiency and Safety of Brentuximab Vedotine as a Salvage Treatment Before Autologous Stem Cell Transplantation in Patients With Relapsed or Refractory Classic Hodgkin Lymphoma: Retrospective Study

**DOI:** 10.1155/ah/3573471

**Published:** 2025-10-12

**Authors:** Kateryna Filonenko, Yana Stepanishyna, Nazar Shokun, Arina Martynchyk, Yevhen Kushchevyi, Iryna Kriachok, Jan Maciej Zaucha

**Affiliations:** ^1^ Department of Hematology and Transplantology, University Medical Center in Gdansk, Gdansk, Poland; ^2^ Hematology Department, Jules Bordet Institute, Brussels, Belgium, bordet.be; ^3^ Bone Marrow Transplantation Department, State Non-Commercial Enterprise “National Cancer Institute”, Kyiv, Ukraine; ^4^ Haematology Department, Olivia Newton John Cancer Research Institute at Austin Health, Melbourne, Australia; ^5^ Department of Hematology and Stem Cell Transplantation, West German Cancer Center Essen University Hospital Essen, Essen, Germany; ^6^ Clinic of Chemotherapy and Oncohematology, State Non-Commercial Enterprise “National Cancer Institute”, Kyiv, Ukraine; ^7^ Department of Hematology and Transplantology, Medical University in Gdansk, Gdansk, Poland

**Keywords:** brentuximab vedotine, high-risk, Hodgkin’s lymphoma, real life, salvage therapy

## Abstract

**Background:**

There is no strong evidence supporting brentuximab vedotine (BV) efficacy as a salvage regimen for relapsed/refractory Hodgkin’s lymphoma (R/R HL) patients before autologous hematopoietic cell transplantation (auto‐HCT).

**Methods:**

We performed multicenter retrospective analysis of efficiency of treatment with the BV monotherapy as salvage regimen versus standard salvage chemotherapy (sCT) in 44 patients with R/R HL from a high‐risk group.

**Results:**

Twenty‐six patients (59.1%) had primary refractory disease. Nineteen patients (43.2%) received BV salvage treatment before auto‐HCT, and 12 (63.6%) achieved complete response (CR) that counts 27.3% of the whole study cohort. There was no difference in the CR rate, 2‐year progression‐free survival, and 2‐year overall survival after BV salvage and sCT (63.2% vs. 60%, respectively, *p* = 0.35; 88.2% vs. 80.7%, *p* = 0.655; 94.1% vs. 100%, *p* = 0.735, respectively). There was no difference in neuropathy of all grades, including Grades 2‐3 between groups.

**Conclusions:**

The BV as a salvage treatment showed quite high efficiency. Despite a worse clinical characteristic of the BV group, there were no significant differences in the outcomes of the auto‐HCT with respect to the salvage regimen (BV versus sCT) used before auto‐HCT. Our results suggest that BV before auto‐HCT in patients refractory to standard 2nd line treatment equalizes their prognosis to the patient sensitive to standard CT with the same tolerance and toxicity.

**Summary**

• Brentuximab vedotine (BV) as salvage therapy before autologous hematopoietic cell transplantation (auto‐HCT) in refractory patients to standard 2nd line treatment has not worsen efficacy and acceptable toxicity.

## 1. Introduction

Despite the very high curative rate in Hodgkin lymphoma (HL), 5% to 30% of patients are refractory or will relapse after the 1st line of treatment, depending on the stage of HL and type of first‐line treatment [1, 2]. The salvage chemotherapy (sCT) followed by auto‐HCT still remains the standard of care, allowing long‐term progression‐free survival (PFS) in about 50% of cases [3].

BV is an antibody–drug conjugate with high efficiency in the treatment of HL and some other types of CD30+ lymphoproliferative tumors. The administration of BV as a consolidation after auto‐HCT for patients with a high risk of relapse will increase the chance for a cure by about 20% [4]. On the other hand, it is known that achievement of complete response (CR) before auto‐HCT improves the 5‐y PFS of about 40% [5]. The present standard of care‐sCT achieves around 25% of CR in the relapsed/refractory (R/R) setting of HL patients. The nonrandomized clinical trials in patients with refractory to the second‐line therapy HL showed 27%–50% CR rates in patients salvaged with BV monotherapy before auto‐HCT and 60%–100% CR rates in patients salvaged with a combination of BV + chemotherapy. The estimated event‐free survival rate in these studies was over 70% when BV is used before auto‐HCT [6–9]. It is recommended by the expert panels that patients refractory to the second line who achieve CR after BV should undergo auto‐HCT and continue BV maintenance as in the AETHERA trial [4]. However, the real‐world data supporting this recommendation are missing. We hypothesize that patients receiving BV before auto‐HCT and who continue BV as consolidation have a similar outcome to those receiving BV only after auto‐HCT. To verify our hypothesis, we analyzed real‐life data of R/R HL from two East European hematological centers. We also present the differences in the approach to R/R HL patients’ treatment in both centers.

## 2. Materials and Methods

This is a multicenter, retrospective analysis of patients treated between 2017 and 2022 in two centers: National Cancer Institute (Kyiv, Ukraine) or University Clinical Center (Gdansk, Poland). We included patients (≥ 18 years old) with R/R classical HL from a high‐risk group (according to the AETHERA trial criteria [4]) who received auto‐HCT and BV consolidation therapy. Data were collected from institutional medical records.

Last‐line salvage treatment before auto‐HCT was either standard sCT or BV in monotherapy according to physician’s choice. The salvage treatments were administered according to the local standard of care. Auto‐HCT was performed in patients who had expected survival of more than 3 months, showed chemosensitive disease or any degree of response, and had no other viable treatment in future without previous auto‐HCT. BV consolidation was administered according to the AETHERA trial criteria [4].

The BV treatment in the Ukrainian center is partially covered within the framework of the Patient Assistance Program supported by Takeda Company. In Poland, BV treatment is reimbursed by the National Health Foundation within the special program. Standard dosing of BV was used in both centers.

The response rates were defined according to the Lugano 2014 criteria, using positron‐emission tomography (PET/CT) [10]. Overall survival (OS) was defined as time from auto‐HCT until the patient’s death, last date of follow‐up, or lost from follow‐up. The primary end point was PFS defined as time from auto‐HCT till the disease progression (PD), patient’s death, or last date of follow‐up. Toxicity was assessed according to the Common Terminology Criteria for Adverse Events (CTCAE) Version 4.0.

Continuous variables were compared using the Mann–Whitney *U* test, and categorical variables with the *χ* ^2^ or Fisher’s exact test. Survival was estimated using the Kaplan–Meier method. Hazard ratios (HRs) with 95% confidence intervals (CI) were calculated using Cox proportional hazard regression.

## 3. Results

**Figure 1:**
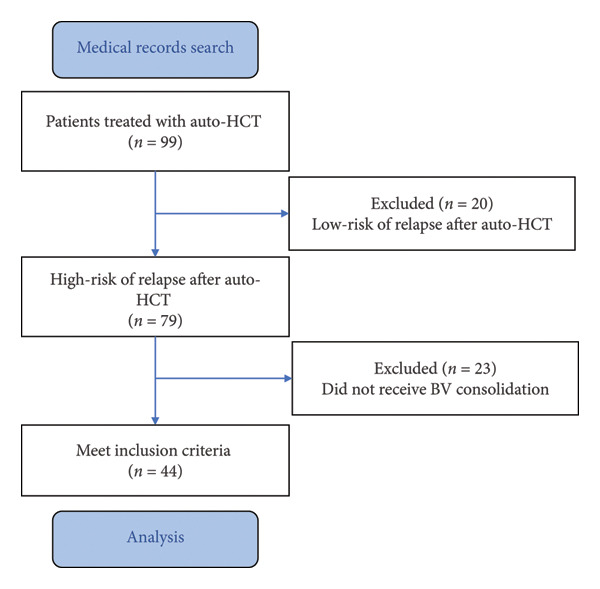
Patient selection flowchart. Auto‐HCT: autologous hematopoietic cell transplantation; BV: brentuximab vedotine.

Ninety‐nine patients with R/R HL, who received auto‐HCT, were identified. Among them, 79 patients (80.0%) had features of high‐risk of relapse after auto‐HCT (Figure 1). Forty‐four patients (44.4%) received BV consolidation, 21 from the National Cancer Institute (Kyiv, Ukraine), and 23 from the University Clinical Center (Gdansk, Poland). The group of patients treated with BV consolidation was the subject of the present analysis.

The median follow‐up period was 24 (1–57) months. The median age of the patients was 33.8 ± 9.8 years, the majority were female (54.5%) patients. The primary refractory disease was registered in 26 patients (59.1%). Nineteen patients (43.2%) received BV salvage treatment before auto‐HCT, and 12 (63.6%) of them achieved CR, while the CR rate in the whole cohort was 27.3%. Median number of treatment lines before auto‐HCT was 2.8 ± 0.96 (2–5). Demographic and clinical characteristics in the general group of patients are presented in Table 1. The median BV cycle before auto‐HCT was 4 (3–8); in total, patients received maximum 16 cycles of BV treatment (in salvage regimen and consolidation). The median number of sCT cycles was 2 (2–4). The most frequently used sCT regimens in Ukrainian population were cisplatin, high‐dose cytarabine, and prednisolone (DHAP), ifosfamide, gemcitabine, and etoposide (IGEV), ifosfamide, carboplatin, and etoposide (ICE), and, in the Polish population, bendamustine, gemcitabine, and dexamethasone (BGD).

**Table 1:** Characteristics of the patients treated either with brentuximab vedotine or standard chemotherapy salvage.

Characteristics	Brentuximab vedotine salvage group (*n* = 19)	Standard salvage chemotherapy group (*n* = 25)	*p*
Age (years), median (range)	34.7 ± 10.6	33.1 ± 9.4	0.61
Gender:			
Male, number (%)	10 (52.6)	10 (40)	0.54
Female, number (%)	9 (47.4)	15 (60)	
Refractory forms, number (%)	10 (52.6%)	16 (64%)	0.54
Number of treatment lines before auto‐HCT	3.5 ± 0.9	2.4 ± 0.7	0.00004
Number of stem cells collected, CD34+ cell × 10^6^/kg body weight	8.2 ± 4.8	10.8 ± 6.9	0.17
Complete response rate before auto‐HCT	12 (63.2)	15 (60)	0.35
Complete response rate after auto‐HCT	18 (94.7)	20 (80)	0.26

Abbreviation: auto‐HCT, autologous hematopoietic‐cell transplantation.

There were no differences in demographic and clinical characteristics in the BV and sCT group, except the number of previous treatment lines. BV was used as salvage treatment later in the treatment course of the patients: median number of previous treatment lines 3.5 in the BV group versus 2.4 in the sCT group, *p* = 0.00004. Nevertheless, the CR rate achieved after BV salvage treatment was similar to the sCT given at earlier stages (63.2% vs. 60%, respectively, *p* = 0.35). There were no differences in the response rates after auto‐HCT, as well as in the collection efficiency in both groups (Table 1).

The 2‐year PFS and OS rates were similar in both groups. The 2‐year PFS was 88.2% (95% CI 60.6%–96.9%) in the BV‐salvage group and 80.7% (95% CI 55.9%–92.5%) in the sCT group; HR 0.9 (95% CI 0.2–5.3), *p* = 0.89. The 2‐year OS was 100% and 94.1% (95% CI 65.0%–99.2%), respectively; HR 1.6 (95% CI 0.1–25.9), *p* = 0.74 (Figure 2).

Figure 2Progression‐free survival (a) and overall survival (b) in patients treated with brentuximab vedotine (BV) versus standard chemotherapy. BV: brentuximab vedotine; OS: overall survival; PFS: progression‐free survival.

**Figure:**
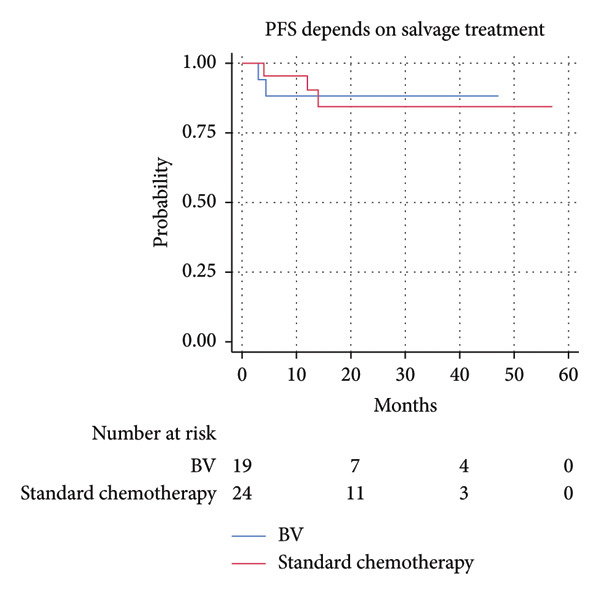
(a)

**Figure:**
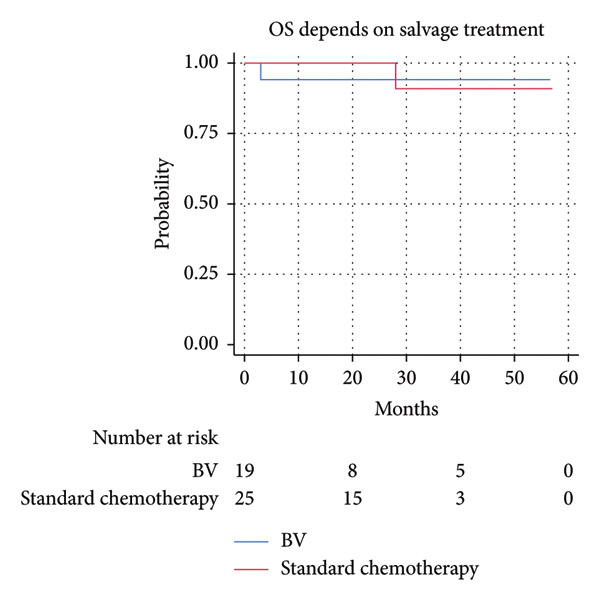
(b)

Two deaths occurred in the study population (one in the BV group and one in the sCT group) due to PD in the Ukrainian patients due to the lack of the treatment options available based on the socioeconomic situation in the country at the moment of the PD confirmation.

Neuropathy of all grades as a BV treatment complication was observed in 9 patients (42.1%) from the BV salvage group; among them, neuropathy of Grades 2 and 3 was observed in 3 (15.8%) patients. In the sCT group, neuropathy was observed in 17 (68%) patients, including Grades 2‐3—in 6 patients (24%), respectively. The difference was not significant (*p* = 0.354). The BV dose was reduced in 6 (31.6%) and 10 (40%) cases, respectively (*p* = 0.753). The treatment discontinuation was observed in 3 patients in each group (16% and 12%), respectively (*p* = 0.753).

Therefore, there were no differences in the immediate and long‐term treatment outcomes in the groups of patients treated with BV‐salvage or sCT regimens. The level of neuropathy, frequency of the dose reduction, and discontinuation of the BV consolidation treatment was similar in both groups. The number of treatment lines was the only factor that was different in compared groups.

Also, we have analyzed patients’ data in the Polish and Ukrainian populations. Detailed description and analysis are available in the Supporting Information (available here). There was no difference in patients’ demographic data. Despite the trend of the higher number of the refractory forms in Polish patients’ population (*p* = 0.07), the CR rate achieved before the auto‐HCT was higher (*p* = 0.0004) with a lower number of the previous treatment lines (*p* = 0.02).

The 2‐year PFS was 94.7% (95% CI 68.1%–99.2%) in the Polish population and 75.9% (95% CI 51.4%–89.2%) in the Ukrainian population (0.19). The 2‐year OS was 100% and 95.2% (95% CI 77.0%–99.3%), respectively (*p* = 0.27).

## 4. Discussion

A key strength of our study is its reflection of real‐world, multicenter data from two academic institutions, which enhance external validity. The inclusion of a defined high‐risk group of R/R HL patients who proceeded to auto‐HCT makes the findings particularly relevant to transplant‐eligible populations. Although retrospective, the structured data collection focused on clinically meaningful endpoints, supporting the clinical relevance of the observations.

The continuous progress is observed in the treatment efficiency in the R/R HL setting. The recently published data from the Center for International Blood and Marrow Transplant Research (CIBMTR) showed an increase in the 3‐year survival probabilities from 72% in 2011–2005 to 92% in 2016–2019 [11]. The success of the last year was possible due to the implementation of the novel drugs, including the BV.

Previously published articles evaluating the efficiency of the BV as salvage therapy in patients who did not respond to multiagent chemotherapy represent retrospective single‐institution data analysis without a comparator. These studies show quite high response rates of the BV salvage treatment (15%–50% of CR rate) and an acceptable safety profile without significant influence on the stem cell harvesting [6–9]. There is a limited amount of data showing treatment efficiency of the BV as a bridge to the auto‐HCT [12, 13].

In our multicenter retrospective study, we have collected data from two reference centers in Poland and Ukraine. We have analyzed the therapy efficiency of BV salvage (bridge to auto‐HCT) versus sCT in high‐risk patients who received BV maintenance.

The majority of patients achieved CR in our study, that is similar or even higher compared to the studies evaluating BV monotherapy as a salvage regimen [6, 7]. Moreover, the BV used at the later stages of the treatment course compared to the sCT achieved CR in patients, who are refractory to sCT. This means that in the real‐world setting, around 25%–30% of the whole R/R patients’ population could be rescued with BV monotherapy.

The survival rates in our patients’ population were comparable to the literature data and confirm the similar results of the BV salvage treatment. Our cohort’s 2‐year PFS and OS for high‐risk R/R HL patients were higher than the survival rates published by Herrera et al. (2018) (77% and 96%, respectively) for patients, who directly proceeded to the auto‐HCT after BV salvage [14].

Similar results were shown in the AMAHRELIS study [15]. This study analyzed treatment results with BV versus sCT in 115 patients with R/R HL who received at least two doses of BV consolidation post‐auto‐HCT. The BV salvage was used in 70% of population, almost twice as much as in our patients’ population (due to earlier reimbursement of the BV in the pretransplant setting). The 2‐year OS and PFS were 96.4% and 75.3%, respectively, with no difference seen in patients with or without previous BV exposure [15].

The neurotoxicity frequency in our study was less than 50% in the BV group and was similar to the sCT group with the majority of events during BV consolidative treatment, which is consistent with the literature data [4, 15].

The one publication showed inferiority of BV salvage in comparison to other regimens, including sCT [16]. The study reported retrospective results of salvage treatment before auto‐HCT with immune check‐point inhibitors (CPIs)–based, BV or sCT in 936 patients (728 patients received sCT) in the period 2011–2020. It showed superiority of CPI‐based regimens (2‐year EFS 79.7%) over all other types of treatment. In comparison of sCT and BV salvage treatment 2‐year EFS was 36.9% vs. 49.6%, respectively, *p* = 0.01 [16].

The abovementioned data support that BV salvage treatment in patients eligible for auto‐HCT is noninferior to the standard of care clinical option, with an acceptable toxicity profile and no negative impact on stem cell collection efficiency.

Our findings should also be considered in the context of emerging immunotherapy regimens, particularly CPIs, which have demonstrated high efficacy in HL treatment. The ongoing shift of BV and CPIs to earlier lines of therapy expands the therapeutic landscape and contributes to higher cure rates in both newly diagnosed and R/R HL patients. Together, these agents form the foundation of modern therapeutic strategies that extend beyond conventional chemotherapy.

Based on the previous data and the appearance of the reimbursement of BV in post‐auto‐HCT consolidation in Poland, investigators from Gdansk carried out a prospective, observational, national study of consolidative BV treatment (BV‐Mazovia, NCT05100056). This will be the first prospective real‐world evidence (RWE) study aiming at assessing outcomes of HL patients in post‐auto‐HCT consolidation. The additional value of the study is inclusion of two subpopulations of patients undergoing consolidation. First, one will be patients salvaged with BV before auto‐HCT and continuing subsequent BV consolidation (so‐called “sandwich approach”). The second subset will be patients undergoing post‐auto‐HCT consolidation salvaged with standard chemotherapy and high risk of relapse (population with characteristics similar to the one in the present AETHERA study). Both approaches are used in clinical practice in Poland.

The surprising data were received during the comparison of the Ukrainian and Polish population. The higher frequency and earlier start of the BV‐salvage treatment could explain the higher CR rates in the Polish population. This hypothesis underlines the immediate necessity of the extension of the diagnostic and treatment possibilities in the healthcare system of Ukraine.

We acknowledge an important limitation of this study related to selection bias, as our analysis included only patients who ultimately underwent auto‐HCT. This design excludes patients who may have experienced insufficient response to salvage therapy, early PD, or treatment‐related complications that precluded transplant, thus potentially overestimating the efficacy of the regimens evaluated. The high CR rate observed in the BV group, for example, may in part reflect this selection effect. However, our primary aim was to examine outcomes in a defined clinical pathway—salvage therapy followed by auto‐HCT and BV consolidation—within the constraints of real‐world practice and national reimbursement policy. However, our primary intent was to analyze outcomes in patients who followed a standardized treatment sequence, including BV‐based salvage, transplant, and BV consolidation. Within this framework, our findings suggest that BV‐containing regimens can induce responses sufficient to transplant and result in post‐transplant outcomes comparable to those achieved with classical chemotherapy‐based salvage approaches. While not designed to assess the overall effectiveness of BV in an unselected population, our data support its role as a viable and effective component of a curative‐intent strategy in transplant‐eligible patients. Future randomized studies are needed to better assess true response rates and long‐term outcomes.

## 5. Conclusion

The BV as a salvage treatment showed noninferior efficiency (CR rate 63%, 2‐year PFS 88%). Despite a worse clinical characteristic of the BV group, there were no significant differences in the outcomes of the auto‐HCT with respect to the salvage regimen (BV versus sCT) used before auto‐HCT. Our results suggest that BV before auto‐HCT in patients refractory to standard second‐line treatment equalizes their prognosis to the patient sensitive to sCT with the same tolerance and toxicity.

## Disclosure

Submission of an article implies that the work described has not been published previously is not under consideration for publication elsewhere, is approved by all authors and tacitly or explicitly by the responsible authorities where the work was carried out, and will not be published elsewhere in the same form if accepted. Part of the data included in this manuscript was presented previously in abstract form at the 49th Annual Meeting of the European Society for Blood and Marrow Transplantation (EBMT), April 23–26, 2023 [17].

## Conflicts of Interest

The authors declare no conflicts of interest.

## Funding

No funding was received for this research.

## Supporting Information

The Supporting Information provides additional information and data related to the comparison of study population based on center localization (Poland and Ukraine). It includes the following:

- Description of treatment approaches in both countries.
- Table S1 with the comparison of patient characteristics.
- Figure S1 with progression‐free survival in the Polish and Ukrainian groups of patients.
- Figure S2 with overall survival in the Polish and Ukrainian groups of patients.

This Supporting Information highlights regional differences in treatment approaches and the importance of improvement of availability of the modern treatments in low–middle income countries.

## Supporting information


**Supporting Information** Additional supporting information can be found online in the Supporting Information section.

## Data Availability

The data that support the findings of this study are available from the corresponding author upon reasonable request.
