# Supplementary material for: Efficiency and Safety of Brentuximab Vedotine as a Salvage Treatment Before Autologous Stem Cell Transplantation in Patients With Relapsed or Refractory Classic Hodgkin Lymphoma: Retrospective Study
Source: Adv Hematol. 2025 Oct 12;2025:3573471. doi: 10.1155/ah/3573471 (PMC12539663; doi:10.1155/ah/3573471)
Supplement: Supplementary file 1 — Supporting Information Additional supporting information can be found online in the Supporting Information section. [file AH-2025-3573471-s001.zip › Filonenko suppl.docx]

*Supplementary materials*

Patients characteristics in both countries populations are presented in the table S1.

Figure S1 and Figure S2 represents comparison of survival curves in both countries populations.

There were some differences in the treatment approaches in both populations. The most frequently used sCT regimens in Ukrainian population were DHAP, IGEV, ICE; in Polish population – BGD (Swoboda et al., 2021). The conditioning regimen LEAM (CCNU, etoposide, Ara-C, melphalan) was used in all patients in Ukrainian center (Sharma et al., 2013). The conditioning regimen BEAM was used in 19 patients (82.6%) and BeEAM (bendamustin, etoposide, Ara-C, melphalan) in 4 patients (17.4%) in Polish center (Gilli et al., 2017). The patients from Polish population were more likely to be transplanted in CR (82.6% versus 38.1%, p=0.0004). The BV salvage treatment was administered earlier in Polish center (median 3^rd^ line versus 4^th^ line in Ukrainian center, p=0.0002). There were no deaths observed in Polish population. One patient is under the diagnosis of the probable secondary lymphoproliferative disease 5 years after auto-HCT. Two patients died in the Ukrainian patients’ population, both due to disease progression in the absence of the further reimbursed treatment.

Table S1. Patient’s characteristics in Ukrainian and Polish population

Figure S1. Progression-free survival in the Polish and Ukrainian groups of patients.

Legend to figure S1. BV – brentuximab vedotine, PFS – progression-free survival, OS – overall survival

Figure S2. Overall survival in the Polish and Ukrainian groups of patients.

Legend to figure S2. BV – brentuximab vedotine, PFS – progression-free survival, OS – overall survival

References

1. Barrington SF, Kirkwood AA, Franceschetto A, Fulham MJ, Roberts TH, Almquist H, Brun E, Hjorthaug K, Viney ZN, Pike LC, Federico M, Luminari S, Radford J, Trotman J, Fossa A, Berkahn L, Molin D, D’Amore F, Sinclair DA, Smith P, O’Doherty MJ, Stevens L, Johnson PW. PET‑CT for staging and early response: Results from the Response‑Adapted Therapy in Advanced Hodgkin Lymphoma study. Blood. 2016;127(12):1531–1538.
2. Sasse S, Bröckelmann PJ, Goergen H, Plütschow A, Müller H, Kreissl S, Buerkle C, Borchmann S, Fuchs M, Borchmann P, Diehl V, Engert A. Long‑Term Follow‑Up of Contemporary Treatment in Early‑Stage Hodgkin Lymphoma: Updated Analyses of the German Hodgkin Study Group HD7, HD8, HD10, and HD11 Trials. Journal of Clinical Oncology. 2017;35(18):1999–2007.
3. Schmitz N, Pfistner B, Sextro M, Sieber M, Carella AM, Haenel M, Dörken B, Berger F, Möller P, Engert A, Diehl V. Aggressive conventional chemotherapy compared with high‑dose chemotherapy with autologous hematopoietic stem-cell transplantation for relapsed chemosensitive Hodgkin's disease: A randomized trial. Lancet. 2002;359(9323):2065–2071.
4. Moskowitz CH, Walewski J, Nademanee A, Masszi T, Agura E, Holowiecki J, Abidi MH, Chen AI, Stiff P, Viviani S, Bachanova V, Sureda A, McClendon T, Lee C, Lisano J, Sweetenham J. Five‑year progression‑free survival from the AETHERA trial of brentuximab vedotin for Hodgkin lymphoma at high risk of progression or relapse. Blood. 2018;132(25):2639–2642.
5. Adams HJ, Kwee TC. Prognostic value of pretransplant FDG‑PET in refractory/relapsed Hodgkin lymphoma treated with autologous stem cell transplantation: Systematic review and meta‑analysis. Annals of Hematology. 2016;95(5):695–706.
6. Moskowitz AJ, Schöder H, Yahalom J, McCall SJ, Fox SY, Gerecitano J, Grewal R, Hamlin PA, Horwitz S, Kobos R, Kumar A, Matasar M, Noy A, Palomba ML, Perales MA, Portlock CS, Sauter C, Shukla N, Steinherz P, Straus D, Trippett T, Younes A, Zelenetz A, Moskowitz CH.PET‑adapted sequential salvage therapy with brentuximab vedotin followed by augmented ifosfamide, carboplatin, and etoposide for relapsed and refractory Hodgkin's lymphoma: A non‑randomised, open‑label, single‑centre, phase 2 study. The Lancet Oncology. 2015;16(3):284–292.
7. Walewski J, Hellmann A, Siritanaratkul N, Ozsan GH, Ozcan M, Chuncharunee S, Goh AS, Jurczak W, Koren J, Paszkiewicz-Kozik E, Wang B, Singh S, Huebner D, Engert A, von Tresckow B.  Prospective study of brentuximab vedotin in relapsed/refractory Hodgkin lymphoma patients not suitable for stem cell transplant or multi‑agent chemotherapy. British Journal of Haematology. 2018;183(3):400–410.
8. Garcia-Sanz R, Sureda A, de la Cruz F, Canales M, Gonzalez AP, Pinana JL, Rodriguez A, Gutierrez A, Domingo-Domenech E, Sanchez-Gonzalez B, Rodriguez G, Lopez J, Moreno M, Rodriguez-Salazar MJ, Jimenez-Cabrera S, Caballero MD, Martinez C. Brentuximab vedotin and ESHAP as highly effective second‑line therapy for Hodgkin lymphoma patients (long‑term results of a trial by the Spanish GELTAMO Group). Annals of Oncology. 2019;30(4):612–620.
9. LaCasce AS, Bociek RG, Sawas A, Caimi P, Agura E, Matous J, Ansell SM, Crosswell HE, Islas-Ohlmayer M, Behler C, Cheung E, Forero-Torres A, Vose J, O'Connor OA, Josephson N, Wang Y, Advani R. Brentuximab vedotin plus bendamustine: A highly active first salvage regimen for relapsed or refractory Hodgkin lymphoma. Blood. 2018;132(1):40–48.
10. Cheson BD, Fisher RI, Barrington SF, Cavalli F, Schwartz LH, Zucca E, Lister TA; Alliance, Australasian Leukaemia and Lymphoma Group; Eastern Cooperative Oncology Group; European Mantle Cell Lymphoma Consortium; Italian Lymphoma Foundation; European Organisation for Research; Treatment of Cancer/Dutch Hemato-Oncology Group; Grupo Español de Médula Ósea; German High-Grade Lymphoma Study Group; German Hodgkin's Study Group; Japanese Lymphorra Study Group; Lymphoma Study Association; NCIC Clinical Trials Group; Nordic Lymphoma Study Group; Southwest Oncology Group; United Kingdom National Cancer Research Institute. Recommendations for initial evaluation, staging, and response assessment of Hodgkin and non-Hodgkin lymphoma: the Lugano classification. Journal of Clinical Oncology. 2014;32(27):3059–3068.
11. Auletta JJ, Kou J, Chen M, Shaw BE. Current use and outcomes of hematopoietic stem cell transplantation: CIBMTR US Summary Slides. CIBMTR. 2021. Available online: <https://cibmtr.org/CIBMTR/Resources/Summary-Slides-Reports> (accessed on 28 April 2023).
12. Chen R, Palmer JM, Martin P, Tsai N, Kim Y, Chen BT, Zain J, Gopal AK, Palmer JM. Results of a Multicenter Phase II Trial of Brentuximab Vedotin as Second‑Line Therapy before Autologous Transplantation in Relapsed/Refractory Hodgkin Lymphoma. Biology of Blood and Marrow Transplantation. 2015;21(12):2136–2140.
13. Kaloyannidis P, Al Zayer M, Al Darweesh M, Al Batran M, Al Garni A, Al Naim A, Al Hashmi H, Kanfar S. Brentuximab vedotin plus bendamustine versus platinum‑based regimens as first salvage therapy and 'bridge' to autologous hematopoietic stem cell transplantation for relapsed/refractory Hodgkin lymphoma. Leukemia & Lymphoma. 2023;64(3):742–745.
14. Herrera AF, Palmer J, Martin P, Armenian S, Tsai NC, Kennedy N, Sahebi F, Cao T, Budde LE, Mei M, Siddiqi T, Popplewell L, Rosen ST, Kwak LW, Nademanee A, Forman SJ, Chen R. Autologous stem-cell transplantation after second-line brentuximab vedotin in relapsed or refractory Hodgkin lymphoma. Annals of Oncology. 2018;29(3):724–730.
15. Marouf A, Cottereau AS, Fouquet G, Kanoun S, Franchi P, Ricci R, Meignan M, Sibon D, Gastinne T, Borel C, Hammoud M, Sicard G, Gille R, Cavalieri D, Bastard AS, Clement-Filliatre L, Lazarovici J, Chauchet A, Fornecker LM, Amorin S, Roquet M, Raus N, Casasnovas RO, Cartron G, Ghesquieres H, Brice P, Hermine O, Bouscary D, Rubio MT, Tamburini J, Fischer BD. Amahrelis: Adcetris maintenance after autologous stem cell transplantation in Hodgkin lymphoma: A real-life study from SFGM-TC and LYSA Groups. Blood. 2020;136(1):20–21.
16. Desai SH, Spinner MA, David K, Bachanova V, Goyal G, Kahl B, Dorritie K, Azzi J, Kenkre VP, Arai S, Chang C, Fusco B, Sumransub N, Hatic H, Saba R, Ibrahim U, Harris EI, Shah H, Murphy J, Ansell S, Jagadish D, Orellana-Noia V, Diefenbach C, Iyenger S, Rappazzo KC, Mishra R, Choi Y, Nowakowski GS, Advani RH, Micallef IN.  Checkpoint inhibitor-based salvage regimens prior to autologous stem cell transplant improve event-free survival in relapsed/refractory classic Hodgkin lymphoma. American Journal of Hematology. 2023;98(3):464–471
17. Filonenko K, Stepanishyna Y, Kushchevyi Y, Martynchyk A. P451 Efficacy and safety of brentuximab vedotin as a salvage treatment before autologous SCT in patients with relapsed and refractory Hodgkin lymphoma – Ukrainian experience. Bone Marrow Transplant*.* 2023;58(suppl 1):460-46. Abstract P451, 49th Annual Meeting of EBMT, 23–26 Apr 2023.
