# Supplementary material for: Efficiency and Safety of Brentuximab Vedotine as a Salvage Treatment Before Autologous Stem Cell Transplantation in Patients With Relapsed or Refractory Classic Hodgkin Lymphoma: Retrospective Study
Source: Adv Hematol. 2025 Oct 12;2025:3573471. doi: 10.1155/ah/3573471 (PMC12539663; doi:10.1155/ah/3573471)
Supplement: Supplementary file 1 — Supporting Information Additional supporting information can be found online in the Supporting Information section. [file AH-2025-3573471-s001.zip › TableS1 - suppl.docx]

Table S1. Patient’s characteristics in Ukrainian and Polish population

| Characteristics | Ukrainian population (n=21) | Polish population (n=23) | p |
| --- | --- | --- | --- |
| Age (years),  median (range) | 31.5±8.1 | 35.9±10.9 | 0.14 |
| Male, number (%) | 8 (38.1) | 12 (52.2) | 0.38 |
| Refractory forms,  number (%) | 9 (42.9) | 17 (73.9) | 0.07 |
| BV salvage, number (%) | 8 (38.1) | 11 (47.8) | 0.56 |
| Number of treatment lines before auto-HCT | 3.2±1.1 | 2.5±0.7 | 0.02 |
| Complete response rate before auto-HCT | 8 (38.1) | 19 (82.6) | 0.0004 |
| Complete response rate after auto-HCT | 17 (81.0) | 22 (95.7) | 0.17 |
| Median follow-up period (range), months | 30 (3-57) | 19 (1-57) | 0.02 |

BV – brentuximab vedotine, auto-HCT – autologous hematopoietic cell transplantation

References

1. Barrington SF, Kirkwood AA, Franceschetto A, Fulham MJ, Roberts TH, Almquist H, et al. PET-CT for staging and early response: results from the Response-Adapted Therapy in Advanced Hodgkin Lymphoma study. Blood. 2016;127(12): 1531-1538.
2. Sasse S, Brockelmann PJ, Goergen H, Plutschow A, Muller H, Kreissl S, et al. Long-Term Follow-Up of Contemporary Treatment in Early-Stage Hodgkin Lymphoma: Updated Analyses of the German Hodgkin Study Group HD7, HD8, HD10, and HD11 Trials. J Clin Oncol. 2017);35(18):1999-2007.
3. Schmitz N, Pfistner B, Sextro M, Sieber M, Carella AM, Haenel M, et al. Aggressive conventional chemotherapy compared with high-dose chemotherapy with autologous haemopoietic stem-cell transplantation for relapsed chemosensitive Hodgkin's disease: a randomised trial. Lancet. 2002;359(9323): 2065-2071.
4. Moskowitz CH,  Walewski J,  Nademanee A,  Masszi T,  Agura E,  Holowiecki J, et al. Five-year PFS from the AETHERA trial of brentuximab vedotin for Hodgkin lymphoma at high risk of progression or relapse. Blood*.* 2018;132(25): 2639–2642.
5. Adams HJ, Kwee TC. Prognostic value of pretransplant FDG-PET in refractory/relapsed Hodgkin lymphoma treated with autologous stem cell transplantation: systematic review and meta-analysis. Ann Hematol. 2016;95(5):695-706.
6. Moskowitz AJ, Schoder H, Yahalom J, McCall SJ, Fox SY, Gerecitano J, et al. PET-adapted sequential salvage therapy with brentuximab vedotin followed by augmented ifosamide, carboplatin, and etoposide for patients with relapsed and refractory Hodgkin's lymphoma: a non-randomised, open-label, single-centre, phase 2 study. Lancet Oncol. 2015;16(3): 284-292.
7. Walewski J, Hellmann A, Siritanaratkul N, Ozsan GH, Ozcan M, Chuncharunee S, et al. Prospective study of brentuximab vedotin in relapsed/refractory Hodgkin lymphoma patients who are not suitable for stem cell transplant or multi-agent chemotherapy. Br J Haematol. 2018;183(3), 400-410.
8. Garcia-Sanz R, Sureda A, de la Cruz F, Canales M, Gonzalez AP, Pinana JL, et al. Brentuximab vedotin and ESHAP is highly effective as second-line therapy for Hodgkin lymphoma patients (long-term results of a trial by the Spanish GELTAMO Group). Ann Oncol. 2019;30(4): 612-620.
9. LaCasce AS, Bociek RG, Sawas A, Caimi P, Agura E, Matous J, et al. Brentuximab vedotin plus bendamustine: a highly active first salvage regimen for relapsed or refractory Hodgkin lymphoma. Blood. 2018;132(1): 40-48.
10. Cheson BD, Fisher RI, Barrington SF, Cavalli F, Schwartz LH, Zucca E, et al. Recommendations for initial evaluation, staging, and response assessment of Hodgkin and non-Hodgkin lymphoma: the Lugano classification. J Clin Oncol. 2014;32(27): 3059-3068.
11. Auletta JJ, Kou J, Chen M, Shaw BE. Current use and outcome of hematopoietic stem cell transplantation: CIBMTR US Summary Slides. In: CIBMTR, Editor 2021. Available online: <https://cibmtr.org/CIBMTR/Resources/Summary-Slides-Reports> (accessed on 28 April 2023).}
12. Chen R, Palmer JM, Martin P, Tsai N, Kim Y, Chen BT, et al. Results of a Multicenter Phase II Trial of Brentuximab Vedotin as Second-Line Therapy before Autologous Transplantation in Relapsed/Refractory Hodgkin Lymphoma. Biol Blood Marrow Transplant. 2015;21(12), 2136-2140.
13. Kaloyannidis P, Al Zayer M, Al Darweesh M, Al Batran M, Al Garni A, Al Naim A, et al. Brentuximab vedotin plus bendamustine versus platinum-based regimens as 1st salvage therapy and 'bridge' to autologous hematopoietic stem cell transplantation for relapsed/refractory Hodgkin lymphoma. Leuk Lymphoma. 2023;64(3): 742-745.
14. Herrera AF, Palmer J, Martin P, Armenian S, Tsai NC, Kennedy N, et al. Autologous stem-cell transplantation after second-line brentuximab vedotin in relapsed or refractory Hodgkin lymphoma. Ann Oncol. 2018;29(3): 724-730.
15. Marouf A,  Cottereau AS,  Fouquet G,  Kanoun S,  Franchi P,  Ricci R, et al. Amahrelis: Adcetris Maintenance after Autologous Stem Cell Transplantation in Hodgkin Lymphoma: A Real Life Study from Sfgmtc and Lysa Groups. Blood*.* 2020;136(1): 20–21.

Desai SH, Spinner MA, David K, Bachanova V, Goyal G, Kahl B, et al. Checkpoint inhibitor-based salvage regimens prior to autologous stem cell transplant improve event-free survival in relapsed/refractory classic Hodgkin lymphoma. Am J Hematol. 2023;98(3):464-471.
