# Supplementary figures and images for: Efficiency and Safety of Brentuximab Vedotine as a Salvage Treatment Before Autologous Stem Cell Transplantation in Patients With Relapsed or Refractory Classic Hodgkin Lymphoma: Retrospective Study
Source: Adv Hematol. 2025 Oct 12;2025:3573471. doi: 10.1155/ah/3573471 (PMC12539663; doi:10.1155/ah/3573471)

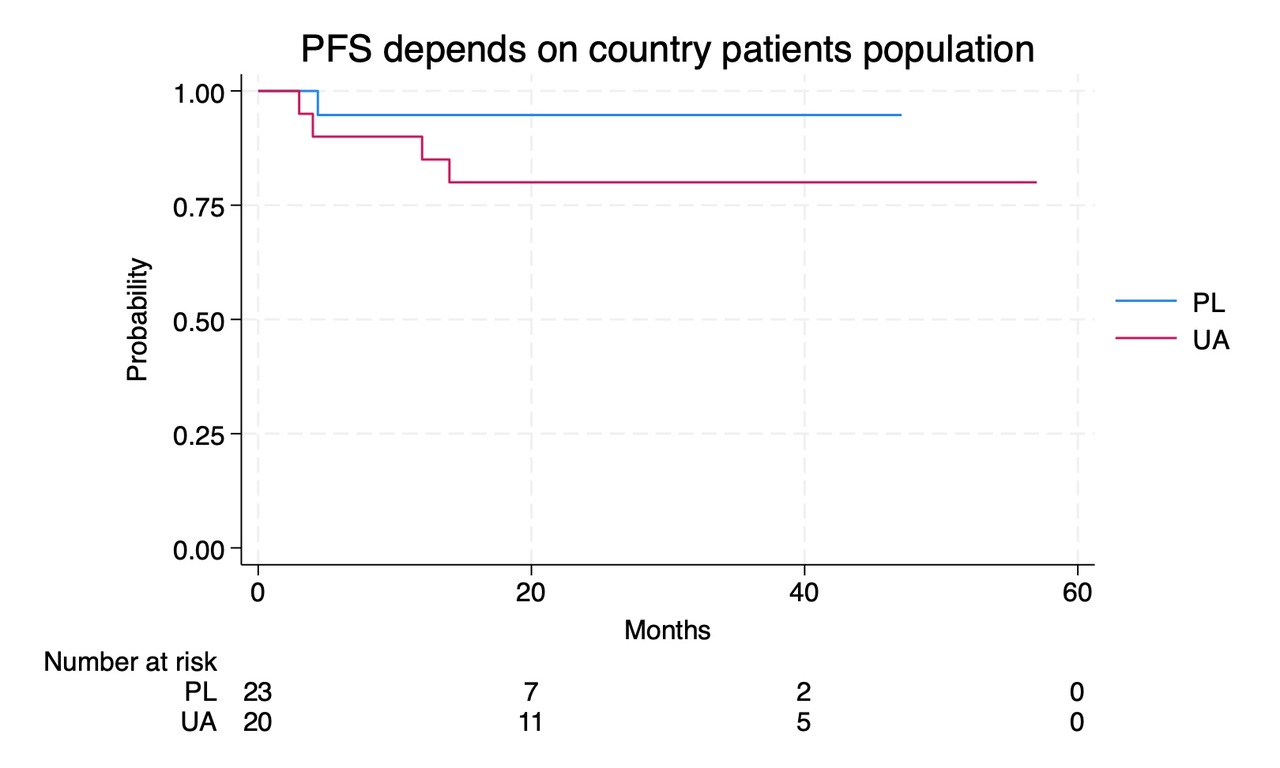

Supplement: Supplementary file 1 — Supporting Information Additional supporting information can be found online in the Supporting Information section. [file AH-2025-3573471-s001.zip › Fig1_supp.png]

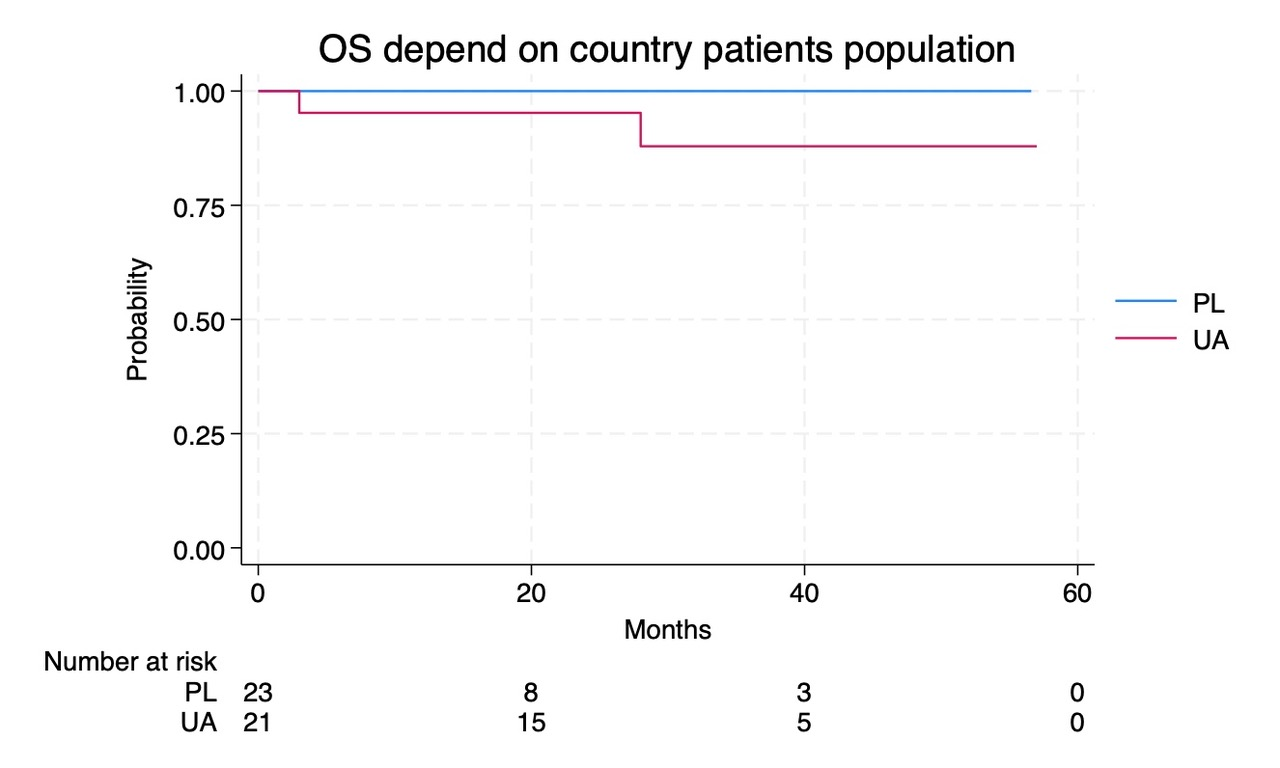

Supplement: Supplementary file 1 — Supporting Information Additional supporting information can be found online in the Supporting Information section. [file AH-2025-3573471-s001.zip › Fig2_supp.png]
